# Hepatitis C Virus in people with experience of injection drug use following their displacement to Southern Ukraine before 2020

**DOI:** 10.1186/s12879-023-08423-5

**Published:** 2023-07-03

**Authors:** Anna Yakovleva, Ganna Kovalenko, Matthew Redlinger, Pavlo Smyrnov, Olga Tymets, Anna Korobchuk, Lyudmyla Kotlyk, Anna Kolodiazieva, Anna Podolina, Svitlana Cherniavska, Petro Antonenko, Steffanie A. Strathdee, Samuel R. Friedman, Ian Goodfellow, Joel O. Wertheim, Eric Bortz, Luke Meredith, Tetyana I. Vasylyeva

**Affiliations:** 1 Medical Sciences Division, University of Oxford, Oxford, UK; 2 Department of Pathology, Division of Virology, University of Cambridge, Cambridge, UK; 3 Department of Biological Sciences, University of Alaska Anchorage, Anchorage, AK USA; 4 Alliance for Public Health, Kyiv, Ukraine; 5 Odesa Regional Virology Laboratory, Odesa, Ukraine; 6 Odesa National Medical University, Odesa, Ukraine; 7 Division of Infectious Diseases and Global Public Health, University of California San Diego, La Jolla, CA USA; 8 Department of Population Health, NYU Grossman School of Medicine, New York, NY USA

**Keywords:** Hepatitis C virus, People who inject drugs, Displacement, Phylodynamics, Nanopore sequencing

## Abstract

**Background:**

Due to practical challenges associated with genetic sequencing in low-resource environments, the burden of hepatitis C virus (HCV) in forcibly displaced people is understudied. We examined the use of field applicable HCV sequencing methods and phylogenetic analysis to determine HCV transmission dynamics in internally displaced people who inject drugs (IDPWID) in Ukraine.

**Methods:**

In this cross-sectional study, we used modified respondent-driven sampling to recruit IDPWID who were displaced to Odesa, Ukraine, before 2020. We generated partial and near full length genome (NFLG) HCV sequences using Oxford Nanopore Technology (ONT) MinION in a simulated field environment. Maximum likelihood and Bayesian methods were used to establish phylodynamic relationships.

**Results:**

Between June and September 2020, we collected epidemiological data and whole blood samples from 164 IDPWID (PNAS Nexus.2023;2(3):pgad008). Rapid testing (Wondfo® One Step HCV; Wondfo® One Step HIV1/2) identified an anti-HCV seroprevalence of 67.7%, and 31.1% of participants tested positive for both anti-HCV and HIV. We generated 57 partial or NFLG HCV sequences and identified eight transmission clusters, of which at least two originated within a year and a half post-displacement.

**Conclusions:**

Locally generated genomic data and phylogenetic analysis in rapidly changing low-resource environments, such as those faced by forcibly displaced people, can help inform effective public health strategies. For example, evidence of HCV transmission clusters originating soon after displacement highlights the importance of implementing urgent preventive interventions in ongoing situations of forced displacement.

**Supplementary Information:**

The online version contains supplementary material available at 10.1186/s12879-023-08423-5.

## Background

Forced migration is associated with worse health outcomes in migrant compared to autochthonous populations, attributed to increased vulnerabilities and hardships experienced throughout the migration process, which are reinforced by structural inequalities, discrimination, and gender-based violence [1]. The burden of otherwise preventable and controllable infectious diseases is often high amongst internally displaced persons (IDPs, persons who have been forced to flee their homes and who have not crossed an internationally recognized border [2]), due at least in part to the collapse of local health systems and infrastructure [3].

Prior to the full-scale Russian invasion of Ukraine in February 2022, Ukraine already had the largest IDPs population in Europe. Over 1.4 million people were internally displaced since the beginning of the war in Donbas (a territory that includes Donetsk and Luhansk administrative regions) and the annexation of Crimea in 2014 [4]. Between 2014 and 2020, over 38,600 IDPs settled in Odesa in southern Ukraine [4].

Concurrently, Ukraine has one of the highest hepatitis C virus (HCV) prevalence levels in the world (3.1% in 2020) facilitated primarily by prevalent injection drug use (IDU) [5]. Although direct-acting antivirals capable of curing HCV infection in > 80% of cases [6] have been available for almost a decade, barriers to access to HCV treatment are especially prominent in low-middle income countries as well as war-torn regions suffering from a breakdown in public health, undermining the feasibility of achieving global HCV elimination by 2030 [7–9].

In people who inject drugs (PWID), national HCV seroprevalence in Ukraine was 56% in 2020: lower amongst PWID in Odesa (47.5%) and Crimea (25.7% – 50.1%, depending on a city) compared to Donetsk (55.2%) and Luhansk (58.4%) [10]. National HCV treatment uptake had risen from 0.11% in 2013 to 6.5% in 2020 – an important step towards HCV elimination—but only 10–20% of those treated were PWID [11]. Although no data on HCV epidemiology in Ukrainian internally displaced PWID (IDPWID) is currently available, IDPs are likely to experience additional barriers in accessing appropriate interventions and treatment options compared to non-displaced populations [3].

Molecular epidemiology, the study of how epidemiological and evolutionary factors shape viral phylogenies, is increasingly utilised in public health settings due to the widespread availability of pathogen genetic sequences and has been used to identify HCV transmission clusters, thus improving our understanding of infection transmission chains [12, 13]. Hard-to-reach mobile populations are often excluded from molecular epidemiology-based HCV surveillance, due to the scarcity of available sequencing data. However, recent advances in portable molecular biology tools applicable in low-resource environments, such as Oxford Nanopore Technology (ONT), have enabled molecular epidemiological characterisation of other viral pathogens such as Ebola and rabies in the field [14, 15].

Herein, we present the first partial and near full length genome (NFLG) HCV sequences from Ukraine, which were sequenced locally as part of a training programme to develop expertise in molecular surveillance efforts. We generated sequences in a field-simulated environment to test field-applicable ONT based NFLG sequencing of HCV, which can be utilised to address current challenges in generating the necessary sequencing data from hard-to-reach mobile populations, such as IDPs. Combining phylodynamic analysis and epidemiological data, we aimed to identify HCV transmission patterns and estimate times of HCV transmission events in IDPWID following displacement. Our innovative approach can help reveal opportunities for improved targeting of local preventative interventions and infection management strategies. Overall, this work expands the potential of molecular epidemiology to the study of the burden of HCV in low-resource and rapidly changing environments, such as those faced by forcibly displaced people.

## Methodology

### Study design and participants

This study was approved by the University of Oxford Tropical Research Ethical Committee (Reference: 530–20). Written informed consent was obtained from all participants. All methods were carried out according to the relevant guidelines and regulations. We used modified respondent-driven sampling (RDS) technique [16] to recruit 164 IDPWID (over 18 years of age) in Odesa, Ukraine, from June to September 2020. At the time of recruitment, all participants undergone a short interview, and were screened for HIV and HCV using two rapid tests, Wondfo® One Step HIV1/2 Whole Blood/Serum/Plasma and Wondfo® One Step HCV Whole Blood/Serum/Plasma. At the same time whole blood samples were collected from all participants using the phlebotomy technique, and serum was isolated at the Odesa Regional Virology Laboratory and stored at ─80 °C. Further information on the study design and participant characteristics is described elsewhere [17].

### Sequencing

Methods were adapted from the ARTIC Network nCoV-2019 Sequencing Protocol V3 LoCost to develop a field-applicable HCV sequencing protocol [18, 19] (https://www.protocols.io/view/ncov-2019-sequencing-protocol-v3-locost-bh42j8ye). Briefly, we employed a 400 bp tiling amplicon scheme with genotype- and subtype-specific primers designed to generate partial or NFLG HCV sequences as per the Primal Scheme protocol using reference strains for HCV genotypes 1a, 1b, and 3a based on most prevalent subtypes in the region [20] (see Supplementary Methods and Supplementary Tables 1 and 2). The obtained cDNA amplicons were purified, pooled, and used for library preparation with the Ligation Sequencing Kit (SQK-LSK109) (Oxford Nanopore Technologies, Oxford, UK). Final libraries were loaded onto new flow cells (FLO-MIN106) (23 samples/flow cell) and sequenced with the MinION device. Consensus sequences from this study have been deposited into GenBank (accession numbers OQ979408-OQ979413, OQ979415-OQ979465). Although MinION based HCV sequencing methods have been described previously [21], we aimed to develop an alternative protocol that meets the need for generation of near real-time sequencing data in field settings, without compromising sample integrity or losing sensitivity. Thus, all sequencing work was carried out in a simulated field environment using a “lab-in-a-suitcase” approach [15, 17]. Further details including bioinformatic workflow, beyond the description in this section, are presented in the Supplementary Materials.

### Phylogenetics

HCV sequences were only included in further phylogenetic analysis if the obtained consensus sequence covered >  = 50% of the reference genome (sensitivity analyses were performed with other coverage levels and are presented in the Supplementary materials). For each identified HCV subtype, all resulting consensus genome HCV sequences were aligned using the Muscle algorithm in AliView [22]. RaxML [23] was then used to reconstruct maximum likelihood (ML) phylogenetic trees for each subtype under a general time-reversible nucleotide substitution model with gamma-distributed rate-variation among sites (GTR + G). We used ClusterPicker [24] to identify possible transmission clusters defined as clades with > 90% bootstrap support and within-clades genetic distance < 3%. Other previously published cluster-defining criteria were considered [12], but did not affect our findings (Supplementary Materials).

We used BEAST 1.10.4 [25] to perform molecular clock analyses and reconstruct population growth history for each of the identified HCV subtypes. We used the Bayesian Skyline population growth model (10 intervals), the GTR + G nucleotide substitution model, and the uncorrelated lognormal relaxed clock model. Since our datasets did not have enough molecular clock signal (estimated by TempEst [26], Supplementary Table 3) due to the samples being contemporaneous, we used previously published estimates for evolutionary rates of HCV subtypes to inform our analyses. Specifically, we used normally distributed priors with mean = 1.48E-3 and standard deviation = 2.3E-4, mean = 1.18E-3 and standard deviation = 2.1E-4, mean = 1.289E-3 and standard deviation = 1.47E-4, for subtypes 1a, 1b, and 3a, respectively [27, 28]. All xml files can be found at https://github.com/HIVMolEpi/HCV_IDPWID.

## Results

### Study population

A total of 164 IDPWID were recruited in Odesa, Ukraine, in June—September 2020 (Table 1). The median participant age was 37 years (range 20–63 years) of whom 18% were female. Any previous HCV testing (rapid or diagnostic tests) was reported by 84.1% (*N* = 138) of participants. A previous positive HCV test was reported by 54.3% (*N* = 75). Only 22.7% (*N* = 17) of participants reporting a previous positive HCV test (*N* = 75), also reported ever receiving treatment for HCV (treatment regimen not reported). Rapid testing in this study identified seroprevalence of HCV of 67.7% (*N* = 111). In addition, 31.1% (*N* = 51) of participants tested positive for both HCV and HIV.

**Table 1:** HCV testing and treatment history

		**N**	**%**
**Previous HCV test**	Yes	138	84.1
	No	26	15.9
**Previous HCV test result**	Positive	75	54.3
	Negative	62	44.9
	Unknown	1	0.7
**Previous HCV treatment received (treatment regimen not reported)**	Yes	17	22.7
	No	58	77.3
**Rapid Test HCV**	Positive	111	67.7
	Negative	53	67.7
**Rapid Test HCV/HIV**	Coinfected	51	31.1

### HCV sequencing data

Of the 164 people recruited, 90 samples resulted in polymerase chain reaction (PCR) amplification producing 57 samples with partial (> 50%) or NFLG sequences, and 33 samples with sequence coverage < 50% (Supplementary Fig. 1). Lower coverage in some samples may be due to low sample quality (samples with degraded RNA), low viral loads, as well as low specificity of primer design due to unavailability of reference genomes from Ukraine. These samples were not included for further analysis and future amplification methods will need to be adapted to improve amplification success rates. Of the samples that failed to amplify (74 or 45.1%), 35 were anti-HCV negative by rapid test, and 39 were anti-HCV positive. Of the 57 partial or NFLG HCV sequences generated (34.8% of the total of 164 samples), genotypes 1a (*N* = 14), 1b (*N* = 23), and 3a (*N* = 20) were identified. Of these, 31 (54.4%) had previously tested positive for HCV but had not received treatment. Of those who had previously tested positive without treatment, five had received a positive anti-HCV test before moving to Odesa. Three out of 57 cases showed evidence of coinfection, i.e., simultaneous infection by multiple HCV subtypes, with genotypes 1a/3a, 1b/3a, and 1b/1a, suggesting the occurrence of multiple exposures to HCV in IDPWID (Supplementary Fig. 2). A total of four participants (7.0% of the total HCV genomes generate) for whom HCV was sequenced and assembled, reported previously being tested positive for HCV and receiving treatment in the past. Further information about treatment history which is unavailable in this study would be necessary to interpret whether these cases represent chronic infection or re-infection. At least 22 of these 57 participants were newly diagnosed with HCV in this study: 11 of them tested negative before and the other 11 have not been tested before.

### HCV phylogenetics and phylodynamics

**Fig. 1:**
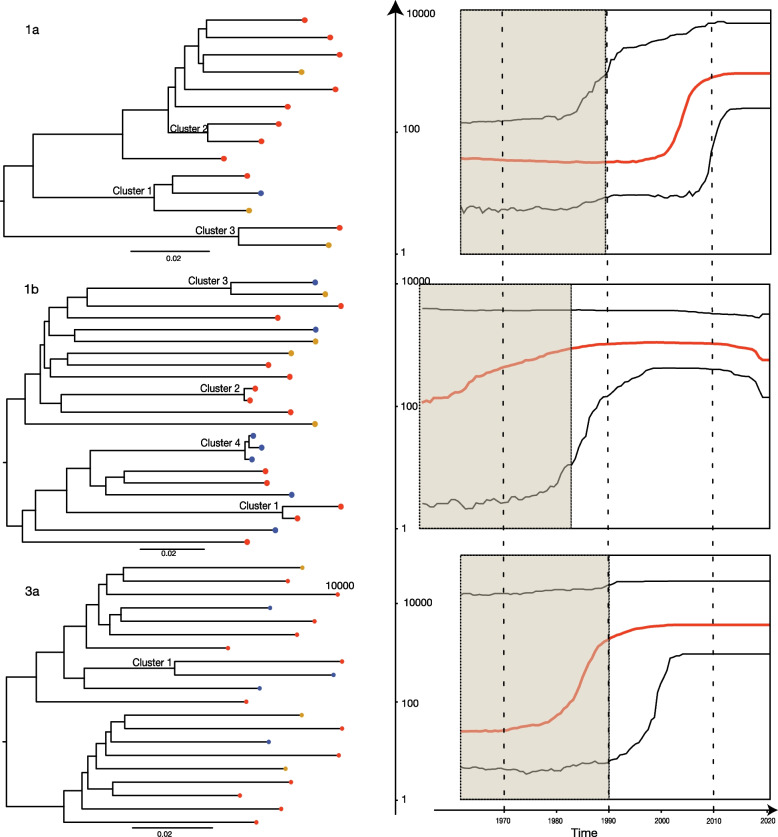
*Left*—Maximum Likelihood phylogenetic trees reconstructed for each HCV genotype and phylogenetic clusters marked on the trees. Tree tips colours correspond to the home regions of IDPWID (red – Donetsk; yellow – Luhansk; indigo – Crimea). Scale bars indicate substitutions/site/year. *Right* – Bayesian Skyline plots reflecting effective population size over time estimated for each of the identified HCV subtypes (red horizontal line – median, black horizontal lines – 95% HPD). Black dotted lines represent the median and lower 95% HPD bound of the time to most recent common ancestor for each subtype. Y-axis indicates log effective population size

**Fig. 2:**
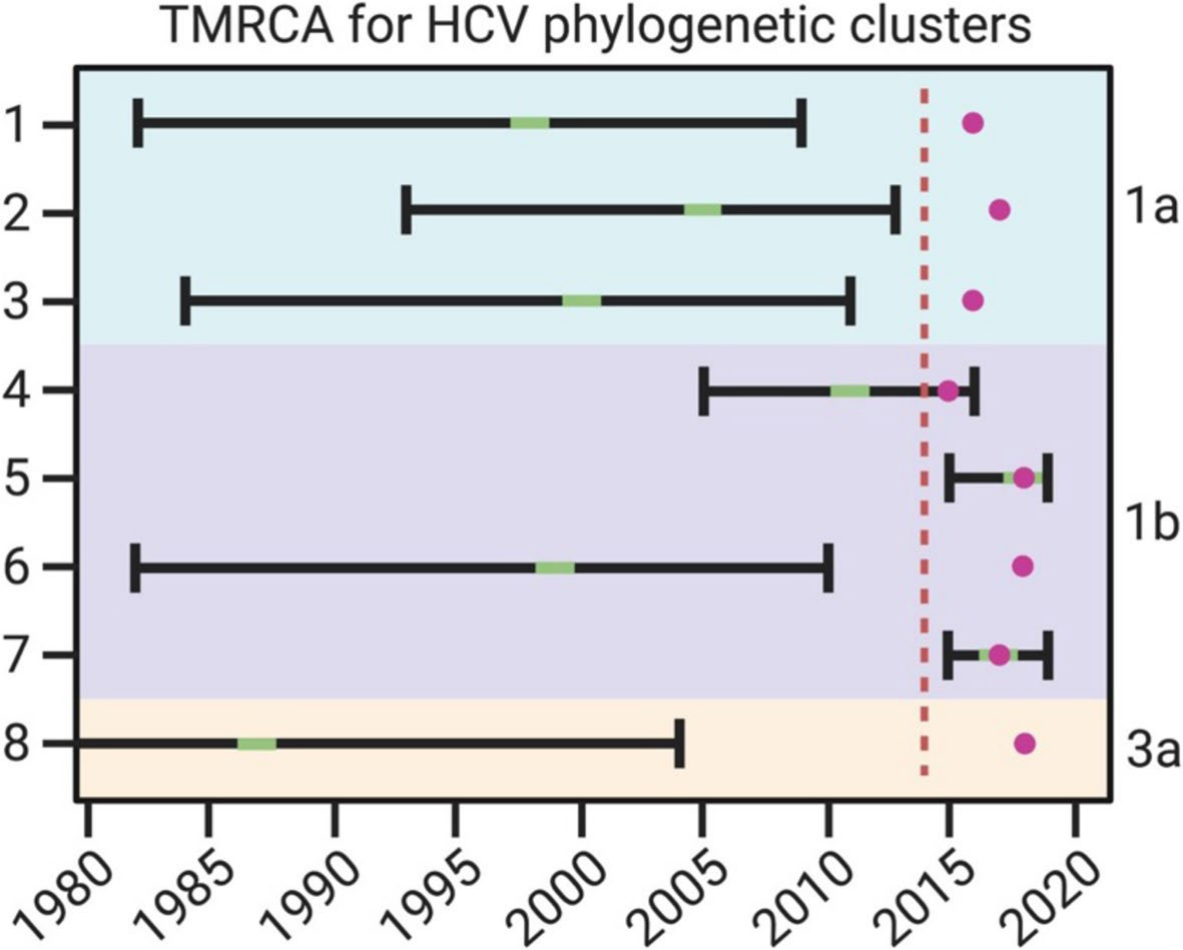
TMRCA uncertainties for HCV phylogenetic clusters in relation to conflict and migration timings. Green indicates TMRCA and black bars are 95% HPD. Purple circle indicates time of latest IDPWID arrival to Odesa within that cluster. Red dashed line indicates the start of the conflict

Phylogenetic trees were reconstructed based on NFLG separately for subtypes 1a, 1b, and 3a and included 14, 23, and 20 sequences, respectively (Fig. 1). We identified three subtype 1a (one triad and two dyads), four subtype 1b (one triad and three dyads), and one subtype 3a (a dyad) potential transmission clusters. Phylodynamic analysis showed that the time of most recent common ancestor (TMRCA) was in 1958 (95% Highest Posterior Density (HPD) 1915—1990), 1949 (1897—1983), and 1955 (1907—1992) for subtypes 1a, 1b, and 3a, respectively. The reconstructed Bayesian Skyline plots showed that the exponential growth of different HCV subtypes likely started in different decades (Fig. 1), with subtype 1b growing before the 1990s, subtype 3a growing before the 2000s, and subtype 1a before the 2010s. The limited number of sequences in our analyses result in wide confidence intervals and prevent further interpretations of the past epidemic growth history. The TMRCAs for all subtype 1a clusters, subtype 1b cluster 6, and the 3a cluster dated pre-conflict and were estimated to be between 1987 and 2013, unlikely capturing a recent transmission event (Table 2 and Fig. 2). Three of the subtype 1b clusters were more recent: TMRCAs for cluster 4, 5, and 7 were estimated to be in November 2011 (May 2005 – April 2016), May 2018 (August 2016—October 2019), and December 2017 (October 2015—May 2019), respectively.

**Table 2:** Cluster composition and time to the most recent common ancestor (TMRCA) for HCV phylogenetic clusters

Subtype	Cluster	Number of sequences	TMRCA	TMRCA 95% HPD	Time of IDPWID arrival to Odesa	Home region
1a	1	3	1998	1982—2009	2014, 2014, 2016	Luhansk, Donetsk, Crimea
	2	2	2005	1993—2013	2015, 2017	Donetsk, Donetsk
	3	2	2000	1984—2011	2016, 2015	Donetsk, Luhansk
1b	4	2	Nov 2011	May 2005—April 2016	May 2015, Jun 2015	Luhansk, Donetsk
	5	2	May 2018	Aug 2016—Oct 2019	Aug 2014, Mar 2018	Donetsk, Donetsk
	6	2	1999	1982—2010	2018, 2017	Luhansk, Crimea
	7	3	Dec 2017	Oct 2015—May 2019	Jun 2015, Sep 2017, Oct 2015	Crimea, Crimea, Crimea
3a	8	2	1987	1961—2004	2018, 2018	Crimea, Donetsk

## Discussion

Here, we present the first HCV NFLG sampled in Ukraine, and show that HCV can be sequenced using Oxford Nanopore MinION in a field-simulated environment. We demonstrate the application of molecular clock analysis to estimate the timing of HCV transmission events relative to the timing of forced displacement, showing evidence that HCV transmission events are likely to occur soon after arrival to the host region.

Of the 57 participants from whom partial or NFLG HCV sequences were available, 29% were found in a total of eight potential transmission clusters. Two of these clusters originated after their members moved to Odesa: in both cases, the upper (older) bound of the TMRCA HPD of the HCV transmission event is after one of the cluster members left the regions of origin, indicating that the transmission happened post-migration. At the same time, the lower (more recent) bound of the TMRCA HPD is within at most one and a half years upon arrival of the last individual to the city. This suggests that transmissions likely happen within a narrow window upon IDPs arrival to host regions and thus prevention efforts in long-term displacement environments will be beneficial as soon after migration as possible. Importantly, the identified HCV clusters are an underestimation of the true number of HCV transmissions in this group and are only indicative of transmission within the IDPWID community.

In both of the identified recent HCV clusters, although transmissions happened after displacement, all cluster members originated from the same home regions (Donetsk and Crimea for clusters 4 and 7, respectively), which supports previous observations of the importance of social networks from the same home community amongst forcibly displaced people [29]. Our phylogeographic analysis of HIV sequences obtained from the same study population showed that upon relocation, the host population is likely to transmit HIV infections to IDPWID [17]. Unfortunately, similar analysis of HCV sequences, or further clustering analysis between migrant, home, and host communities, was not possible due to a lack of available genetic sequence data from autochthonous populations, despite Ukraine having one of the highest HCV burdens in the world [5, 20]. At present, only one partial HCV genetic sequence from Ukraine is available in the GenBank database, collected in 2020 (GenBank Accession Number: OM939210). Inclusion of this sequences in our analysis was not possible as it has no additional epidemiological information available.

Furthermore, the wide HPD intervals for these analyses, specifically when estimating the age of the tree, or TMRCA, are a limitation of the study. This is because our sample size is very low when analysing each subtype separately as required for this analysis, and because all our samples are contemporaneous, sampled within a few months. Lack of older publicly available HCV genomes from Ukraine makes it impossible to get a narrower estimate.

Previous reports of genotype distribution in the general population in Ukraine in the early 2010s showed low prevalence of HCV infection with subtype 1a (1.6%), and a high prevalence of 1b (42.1%), followed by genotype 3 (28.8%) [20]. In this study, we report much higher prevalence of HCV infection with the 1a (25%) subtype, and comparable levels of infection with 1b (40%) and 3a (35%) within IDPWID in Odesa. The HCV subtype 1b dominates epidemics in the neighboring Russian Federation (1a – 2.1%; 1b – 52.8%; 3 – 36.3%) and several Central Asian countries, such as Uzbekistan and Azerbaijan, whilst 1a is more commonly found in western Europe [20]. Higher 1a prevalence amongst HIV co-infected patients compared to the general population has been linked to IDU mediated transmission elsewhere [30], and thus is expected in our study population, where HIV/HCV coinfection was prevalent. The increase in 1a prevalence in our study, reflected in the most recent growth of this subtype compared to subtypes 1b and 3a as estimated by our reconstructed Bayesian Skyline plots, follows the increase in HIV transmission in PWID [31].

Our results suggest that HCV seroprevalence in IDPWID residing in Odesa in 2020 (67.7%) is higher than the prevalence reported in 2015 in both autochthonous PWID (47.5%) and in PWID residing in Donetsk (55.2%) [10]. This may reflect our recruitment strategy, given the enhanced coupon distribution for HIV-positive participants who were not in ART treatment at the time of enrolment [17]. Of those previously reporting a positive anti-HCV test result, 22% had also received treatment in the past (treatment regimen not reported), compared to only 9.4% receiving treatment in a 2013 study, which may be due to a recent increase in targeted HCV treatment efforts for the PWID community in the area [32, 33]. We were also unable to generate HCV genomes from 39 HCV seropositive samples, potentially indicating successful treatment, spontaneously cleared infections, or poor sample quality (samples with degraded RNA) amongst other reasons detailed in Supplementary Fig. 1. Conversely, we generated four HCV genomes from IDPWID who previously received treatment, of which two were found in potential transmission clusters, likely indicating a re-infection or an incomplete treatment course. Continued engagement in treatment is crucial for transmission and disease progression prevention, but forcibly displaced people might face additional barriers in accessing these services [1, 3].

Many IDPWID in our sample initiated IDU after the beginning of the conflict (30.2%) or even after they migrated to Odesa (14.8%). Substance use amongst forcibly displaced people as a coping mechanism following conflict, violence, and migration stress is well documented in current literature [34]. In our study, two individuals who reported starting IDU after migration were found in HCV transmission clusters with those reporting 15 to 20 years IDU experience. Although IDPWID with prior experience of IDU may be more likely to seek and engage with harm reduction programs, efforts to inform and engage new injectors immediately following displacement should be prioritised to reduce HCV transmission amongst this population.

## Conclusions

Forced displacement is a highly heterogenous and complex phenomenon, and is dependent on multiple factors including historical, economic, social, and political landscapes. The displacement journey may differ across time: exposure to risk factors and hardships may continuously evolve. We show that migration data coupled to phylodynamic analysis can help resolve cluster characteristics and transmission timings amongst forcibly displaced people, which to the best of our knowledge has not yet been assessed for HCV transmission [35, 36]. Regions with large displaced populations, limited laboratory capacities, and high prevalence of infectious diseases can especially benefit from the proposed approach to inform the most effective time to implement preventative intervention for this high-risk group [37].

## Supplementary Information

**Additional file 1.** Supplementary Methods and Figures.

**Additional file 2: Supplementary Table 1.** Accession numbers for reference genomes used for HCV primer design. **Supplementary Table 2.** HCV Sequencing Primers. **Supplementary Table 3.** Results of the TempEst analysis. **Supplementary Table 4.** Accession numbers of reference genomes used for HCV genome assembly.

## Data Availability

All genetic sequences generated in this study are available on GenBank (accession numbers OQ979408-OQ979413, OQ979415-OQ979465). For phylogenetic analysis, all xml files can be found at https://github.com/HIVMolEpi/HCV_IDPWID.

## Abbreviations

IDP: Internally displaced persons
HCV: Hepatitis C virus
IDU: Injection drug use
PWID: People who inject drugs
IDPWID: Internally displaced people who inject drugs
ONT: Oxford Nanopore Technology
NGLG: Near full length genome
TMRCA: Time of most recent common ancestor
