# Supplementary material for: Hepatitis C Virus in people with experience of injection drug use following their displacement to Southern Ukraine before 2020
Source: BMC Infect Dis. 2023 Jul 3;23:446. doi: 10.1186/s12879-023-08423-5 (PMC10316605; doi:10.1186/s12879-023-08423-5)
Supplement: Supplementary file 1 — Additional file 1. Supplementary Methods and Figures. [file 12879_2023_8423_MOESM1_ESM.pdf]

Supplementary Methods

RNA extraction and cDNA synthesis

Viral RNA was extracted from 140 µL serum using QIAamp Viral RNA Minikit (Qiagen), following the manufacturer's instructions. Extracted RNA was eluted in a 60 µL volume, aliquoted and stored until use at ─80°C. Extracted RNA was reverse transcribed using SuperScript IV Reverse Transcriptase (SSIV RT, ThermoFisher Scientific). Each reaction included 1 µL random hexamers (ThermoFisher Scientific), 1 µL 10 mM dNTP (ThermoFisher Scientific), 3 µL SSIV buffer, 0.5 µL 0.1M DTT, 0.5 µL RNaseOUT (ThermoFisher Scientific), 0.5 µL SSIV RT and 6.5 µL RNA extract. The mix was incubated at 23°C for 10 min, 50°C for 50 min, 75°C for 10 min, and then held at 4°C.

Primer design

To amplify and sequence low abundance viruses directly from the clinical samples we employed a tiling amplicon scheme with the genotype- and subtype-specific primers. Primers were designed to generate near full length HCV genome sequences as per the Primal Scheme (https://primalscheme.com) protocol based on multiplex tiling PCR amplification of 400bp amplicons overlapping neighboring amplicons by 75 nt[1]. A collection of reference strains for HCV genotypes 1a, 1b, and 3a were identified based on closest available geographical and temporal sequences from GenBank (Supplementary Table 1). Primers for each genotype were pooled into 100 µM pool A and B stock mixes based on alternate regions, which do not overlap within the same pool (Supplementary Table 2).

Amplification

HCV PCR amplification and sequencing methods were adapted from the ARTIC Network nCoV-2019 Sequencing Protocol V3 LoCost (https://www.protocols.io/view/ncov-2019-sequencing-protocol-v3-locost-bh42j8ye). Briefly, PCR amplifications were performed using Q5 Hot Start High-Fidelity DNA Polymerase (NEB). Two separate reactions for primer pool A and B were prepared in a final volume of 12.5 µL, containing 2.5 µL 5X Q5 Reaction Buffer, 0.25 µL Q5 Hot Start DNA Polymerase, 10 µM primer pool (A or B), 0.5 µL 10 mM dNTPs, 3.75 µL nuclease-free water and 3.75 µl cDNA template. Thermocycling was performed using a mini16 thermal cycler (minipcr) with the following program: 98°C for 30 secs, then 35 cycles of 98°C for 15 secs, 62.5°C for 5 min, and final hold at 4°C. Pool A and B reaction products for a given sample were pooled together in equal volumes and purified with SPRI beads (x1 ratio) followed by elution in 25 µL of nuclease-free water. Samples were quantified QuantiFluor® ONE dsDNA Dye (Promega) using the Quantus Fluorometer (Promega).

Oxford Nanopore Library Preparation and Sequencing

Amplicon ends were prepared for ligation with the NEBNext Ultra II End Repair/dA-Tailing Module (NEB). Unique barcodes from the Oxford Nanopore Native Barcoding Expansion kits EXP-NBD104 (1-12) and EXP-NBD114 (13-24) were ligated to each sample using the NEBNext Ultra II Ligation Module (NEB). Following barcoding, reactions were pooled together in equal volumes and purified with SPRI beads (x0.4 ratio). Finally, Oxford Nanopore sequencing adapters were ligated with the NEBNext Quick Ligation Module (NEB) and the library was purified with SPRI beads again (x1 ratio) and quantified using the Quantus Fluorometer (Promega). Final libraries were loaded onto a flow cell (FLO-MIN106) and sequenced with the MinION.

Bioinformatic workflow

All runs were basecalled using guppy basecaller 4.4.2 with the high accuracy model. Demultiplexing was performed as a separate step with guppy barcoder using additional arguments to detect mid strand barcodes. During the demultiplexing step barcodes and an additional 25nt were trimmed from the reads to remove primers. Reads were aligned to 54 HCV reference genomes with Minimap2[2] and the reference with the highest number of primary mappings as determined by samtools[3] was used as a draft genome for a reference guided assembly (Supplementary Table 4). Racon 1.4.21[4] was used to polish the reference genome with the no trim argument enabled and window size set to 300nt. The draft generated by racon was then polished with medaka consensus (https://github.com/nanoporetech/medaka) and any nucleotides with less than 20x coverage were masked. To test for the presence of multiple genomes within the sample, reads that mapped to the previously generated assembly were removed and the remaining reads were again aligned to the 54 reference genomes. Barcodes with over 200k reads mapped to a second genome were used to generate additional assemblies.

Sensitivity analysis

Previously published cluster-defining criteria were considered[5,6]. For subtype 1a analysis, the number of identified clusters was the same if the genetic distance threshold ranged between 3-6%. If the bootstrap support was relaxed to 80 instead of 90 and genetic distance threshold was considered at 4.5%, then 1 additional cluster of 4 sequences was identified. For subtypes 1b and 3a analyses, the same clusters were identified with genetic distance threshold varying 3-6% and bootstrap support of 80.

Supplementary Methods References

1. Quick, J., et al., *Multiplex PCR method for MinION and Illumina sequencing of Zika and other virus genomes directly from clinical samples.* Nature protocols, 2017. **12**(6): p. 1261-1276.
2. Li, H., *Minimap2: pairwise alignment for nucleotide sequences.* Bioinformatics, 2018. **34**(18): p. 3094-3100.
3. Li, H., et al., *The Sequence Alignment/Map format and SAMtools.* Bioinformatics, 2009. **25**(16): p. 2078-2079.
4. Vaser, R., et al., *Fast and accurate de novo genome assembly from long uncorrected reads.* Genome Res, 2017. **27**(5): p. 737-746.
5. Falade-Nwulia, O., et al., *Factors associated with phylogenetic clustering of hepatitis C among people who inject drugs in Baltimore.* BMC Infectious Diseases, 2020. **20**(1): p. 815.
6. Bradshaw, D., et al., *Transmission of hepatitis C virus in HIV-positive and PrEP-using MSM in England.* Journal of Viral Hepatitis, 2020. **27**(7): p. 721-730.

Supplementary Figures

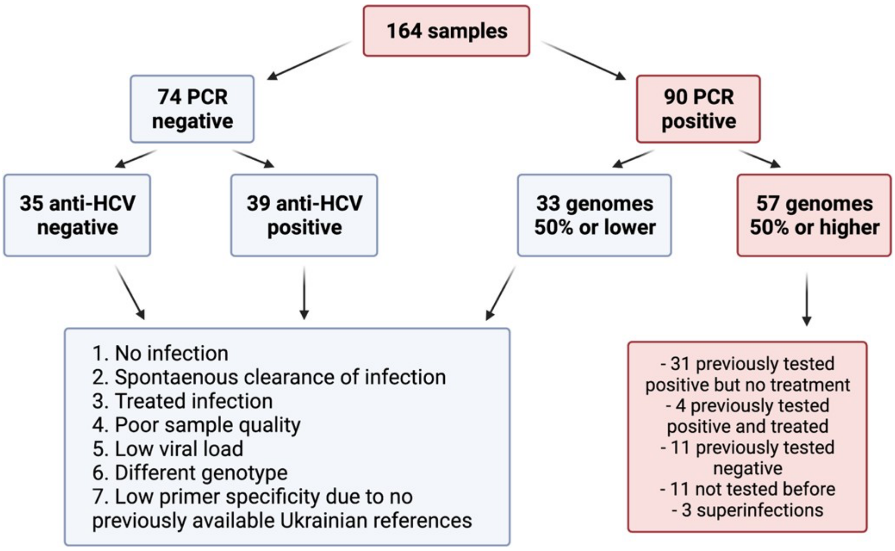

Supplementary Figure 1

Sequencing flow diagram. Number of samples and their characteristics which were successfully amplified by polymerase chain reaction (PCR) and sequenced producing genome lengths 50% or higher are indicated in red boxes. Number of samples and the reasons behind a negative amplification result, or sequences resulting in less than 50% of the genome are indicated in blue boxes.

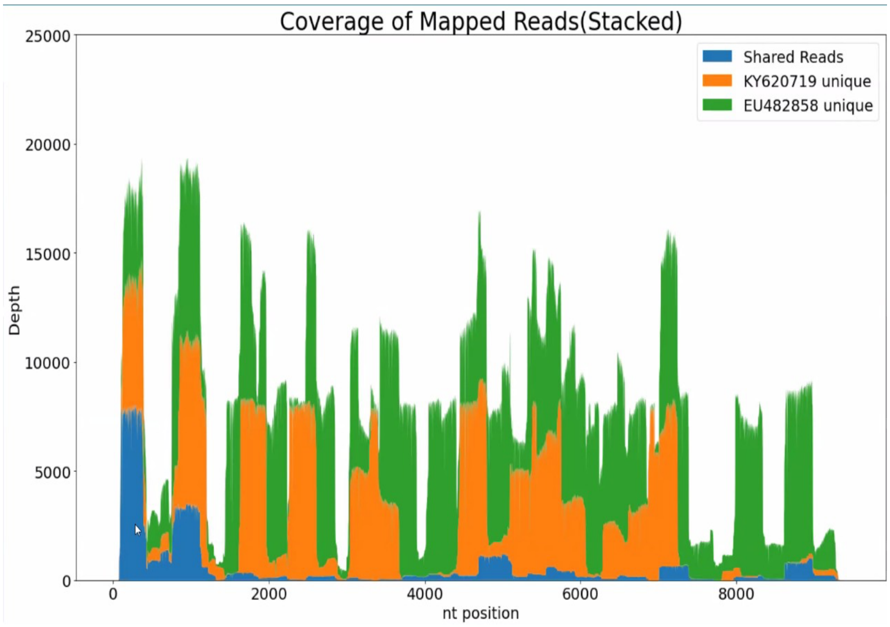

Supplementary Figure 2

Long read tiling primer assembly showing coverage (read depth, y-axis) of HCV genome (nt, x-axis) for example case #01-02. Coinfection was analysed by successive masking and reference-based assembly to two HCV subtypes 1a (Accession EU482858) and 3a (KY620719).
